# Geographical Distribution, Incidence, Malignancies, and Outcome of 136 Eastern Slavic Patients With Nijmegen Breakage Syndrome and *NBN* Founder Variant c.657_661del5

**DOI:** 10.3389/fimmu.2020.602482

**Published:** 2021-01-08

**Authors:** Svetlana O. Sharapova, Olga E. Pashchenko, Anastasiia V. Bondarenko, Svetlana S. Vakhlyarskaya, Tatjana Prokofjeva, Alina S. Fedorova, Ihor Savchak, Yuliya Mareika, Timur T. Valiev, Alexander Popa, Irina A. Tuzankina, Elena V. Vlasova, Inga S. Sakovich, Ekaterina A. Polyakova, Natalia V. Rumiantseva, Irina V. Naumchik, Svetlana A. Kulyova, Svetlana N. Aleshkevich, Elena I. Golovataya, Nina V. Minakovskaya, Mikhail V. Belevtsev, Elena A. Latysheva, Tatiana V. Latysheva, Alexander G. Beznoshchenko, Hayane Akopyan, Halyna Makukh, Olena Kozlova, Dzmitry S. Varabyou, Mark Ballow, Mei-Sing Ong, Jolan E. Walter, Irina V. Kondratenko, Larysa V. Kostyuchenko, Olga V. Aleinikova

**Affiliations:** ^1^ Research Department, Belarusian Research Center for Pediatric Oncology, Hematology and Immunology, Minsk, Belarus; ^2^ Immunology Department, Pirogov Russian National Research Medical University, Moscow, Russia; ^3^ Department of Pediatric Infectious Diseases and Pediatric Immunology, Shupyk National Medical Academy of Postgraduate Education, Kiev, Ukraine; ^4^ Clinical Immunology and Rheumatology Department, Russian Children’s Clinical Hospital of Pirogov Russian National Research Medical University, Moscow, Russia; ^5^ Pediatric Clinic, Children’s Clinical University Hospital, Riga, Latvia; ^6^ Pediatric Department, West-Ukrainian Specialized Children’s Medical Center, Lviv, Ukraine; ^7^ Chemotherapy Hemoblastoses Department, Pediatric Oncology and Hematology Research Institute of N.N. Blokhin National Cancer Research Center of the Ministry of Health of Russian Federation, Moscow, Russia; ^8^ Propedevtica of Childhood Diseases Faculty, Pirogov Russian National Research Medical University, Moscow, Russia; ^9^ Institute of Immunology and Physiology of the Branch of the Russian Academy of Sciences, Federal State Autonomous Educational Intuition of Higher Professional Education (Ural Federal University of a Name of the First President of Russia, B.N. Yeltsin), Yekaterinburg, Russia; ^10^ Clinical Department, Regional Children’s Clinical Hospital №1, Yekaterinburg, Russia; ^11^ Research Department, Republican Medical Center (Mother and Child), Minsk, Belarus; ^12^ Pediatric Oncology Department, N.N. Petrov National Medical Research Center of Oncology, St-Petersburg, Russia; ^13^ Immunopathology Department, NRC Institute of Immunology FMBA, Moscow, Russia; ^14^ Department of Pediatric Oncology/Hematology, Regional Children’s Hospital, Ryazan, Russia; ^15^ Institute of Hereditary Pathology of National Academy of Medical Sciences of Ukraine, Lviv, Ukraine; ^16^ West-Ukrainian Specialized Children’s Medical Center, Lviv, Ukraine; ^17^ Department of Ecologic Geography, Belarusian State University, Minsk, Belarus; ^18^ Department of Pediatrics, University of South Florida at Johns Hopkins All Children’s Hospital, Saint Petersburg, FL, United States; ^19^ Department of Population Medicine, Harvard Medical School, Harvard Pilgrim Health Care, Boston, MA, United States; ^20^ Department Pediatric Allergy/Immunology, University of South Florida at Johns Hopkins All Children’s Hospital, Saint Petersburg, FL, United States

**Keywords:** Nijmegen breakage syndrome (NBS), founder variants, incidence in East Slavs, lymphomas, risk of malignancies, geographical location, social adaptation, quality of life

## Abstract

Nijmegen breakage syndrome (NBS) is a DNA repair disorder characterized by combined immunodeficiency and a high predisposition to lymphoid malignancies. The majority of NBS patients are identified with a homozygous five base pair deletion in the *Nibrin* (*NBN)* gene (c.657_661del5, p.K219fsX19) with a founder effect observed in Caucasian European populations, especially of Slavic origin. We present here an analysis of a cohort of 136 NBS patients of Eastern Slav origin across Belarus, Ukraine, Russia, and Latvia with a focus on understanding the geographic distribution, incidence of malignancy, and treatment outcomes of this cohort. Our analysis shows that Belarus had the highest prevalence of NBS (2.3 per 1,000,000), followed by Ukraine (1.3 per 1,000,000), and Russia (0.7 per 1,000,000). Of note, the highest concentration of NBS cases was observed in the western regions of Belarus and Ukraine, where NBS prevalence exceeds 20 cases per 1,000,000 people, suggesting the presence of an “Eastern Slavic NBS hot spot.” The median age at diagnosis of this cohort ranged from 4 to 5 years, and delay in diagnosis was more pervasive in smaller cities and rural regions. A total of 62 (45%) patients developed malignancies, more commonly in males than females (55.2 vs. 34.2%; *p*=0.017). In 27 patients, NBS was diagnosed following the onset of malignancies (mean age: 8 years). Malignancies were mostly of lymphoid origin and predominantly non-Hodgkin lymphoma (NHL) (*n*=42, 68%); 38% of patients had diffuse large B-cell lymphoma. The 20-year overall survival rate of patients with malignancy was 24%. However, females with cancer experienced poorer event-free survival rates than males (16.6% vs. 46.8%, *p*=0.036). Of 136 NBS patients, 13 underwent hematopoietic stem cell transplantation (HSCT) with an overall survival of 3.5 years following treatment (range: 1 to 14 years). Indications for HSCT included malignancy (*n*=7) and immunodeficiency (*n*=6). Overall, 9% of patients in this cohort reached adulthood. Adult survivors reported diminished quality of life with significant physical and cognitive impairments. Our study highlights the need to improve timely diagnosis and clinical management of NBS among Eastern Slavs. Genetic counseling and screening should be offered to individuals with a family history of NBS, especially in hot spot regions.

## Introduction

Nijmegen breakage syndrome (NBS) is a syndromic combined immunodeficiency with an autosomal-recessive inheritance. It is a chromosome instability disorder with hypersensitivity to ionizing radiation and aberrant cell-cycle checkpoint control and, therefore, high susceptibility to lymphoid malignancies. Patients experience growth delay and developmental defects with visible stigmata of severe and progressive microcephaly, a distinct facial appearance and lack of secondary sex characteristics in females. The disease is caused by pathogenic variants in the *NBN* gene, which encodes nibrin, a component of the complex involved in the cellular response to DNA double-strand breaks (1–3).

Most patients with NBS originate from Central and Eastern Europe with a Slavic background, and there is an accumulation of patients in Poland, Southeast Germany, Czech Republic, Ukraine, and Russia (3–5). Sporadic cases have been published in patients of Middle Eastern origin (6, 7). The majority of NBS patients are identified with a homozygous five base-pair deletion in the *NBN* gene (c.657_661del5, p.K219fsX19) with a founder effect observed in Caucasian European populations, especially of Slavic origin (8). Increased morbidity and mortality in this cohort are related to severe infections (in more than half of patients), autoimmune complications (in up to 1/3 of patients), and malignancies (in about half of patients) under the age of 20 years (9, 10).

Despite recent publications on the clinical manifestations, immune abnormalities, and malignancies in patients with NBS, a number of unresolved questions remain (8–10). The indication for hematopoietic stem cell transplantation (HSCT) for primary immunodeficiency with or without malignancy is not well defined in NBS. Mental health and psychosocial challenges may also contribute to poor quality of life; however, there remains a paucity of published data addressing these concerns. Furthermore, family planning to prevent the birth of children with NBS in geographic locations with the founder variant has not been widely implemented.

In this report, we present data on a large NBS cohort of genetically confirmed East Slavic patients with a specific focus on geographic case distribution, incidence, oncological complications, and outcome of treatment, including HSCT. In addition, we investigate the quality of life and social adaptation of adult survivors. The overall goal of our paper is to raise awareness of additional NBS “hot spots” in Eastern Europe, beyond the Polish region, and shed light on current medical and social challenges in this vulnerable and still underserved population.

## Material and Methods

### Patients

We conducted a multicenter retrospective study enrolling 136 NBS patients of Eastern Slavic origin from Belarus, Ukraine, Russia, and Latvia (Russian origin of patients).

### Ethics Statement

Informed consent forms were signed by the parents as requested and approved by the institutional review boards of local institutions involved. The protocol of study was approved by the institutional review board of the Belarusian Research Center for Pediatric Oncology, Hematology, and Immunology (IRB protocol #0011715). Written informed consent for publishing images of patients was obtained from each patient’s parents.

### Study Design

A detailed questionnaire was administered to the treating physicians to collect the following patient information: demographics (gender, country, place and year of birth), history of malignancy (primary and secondary cancer incidence, treatment, transplantation), and overall survival (OS). We estimated the prevalence of NBS in each country under study using data from our study as well as published data, whereby the numerator was the number of cases detected and the denominator was the number of live births during the reported study period. The number of live births in Ukraine and Belarus were calculated based on reports from Ukrainian and Belarusian State Statistics Services, respectively (http://database.ukrcensus.gov.ua and https://www.belstat.gov.by). We further divided each country into Western and Eastern regions and compared the prevalence of NBS in these regions. Additionally, we estimated the prevalence of NBS in the West Slavs (Poland, Czech Republic, Slovakia) using published data and compared the prevalence of NBS in the East and West Slavs.

To examine the impact of the disease on patients’ quality of life (QoL), we conducted a telephone survey with adult survivors (*n*=11). Long-term social adaptation, an aspect of QoL, was evaluated in terms of the attainment of adult life goals relating to education, employment, and relationships (11). In addition, we assessed patients’ self-reported QoL using the Medical Outcomes Short Form-36 (SF-36) health survey (12). The survey tool comprises 36 questions and yields an 8-scale profile of scores as well as summary measures of physical and mental health, known as the physical (PCS) and mental component summary (MCS) measures. These eight domains can be categorized into higher order clusters of physical health (physical function, role–physical, bodily pain, general health) and mental health (vitality, social function, role–emotional, and mental health). Lower PCS and MCS scores reflect lower QoL relating to physical and mental health status, respectively. These measures have been used widely in health surveys (12). For each patient surveyed, we quantified the whole score for both the PCS and MCS and categorized clusters.

### Mapping of Variants

Mapping was performed by the* ArcGIS 10.5* program on the map of Eastern Europe and Russia. The birthplace of patients is indicated by the location of the blue circles.

### Statistical Analysis

Descriptive statistical analyses were carried out for all scores of the SF-36 QoL. We quantified the OS of our cohort, defined as the time from diagnosis to death from any cause based on data obtained on the date of last follow-up. We further estimated event-free survival (EFS) rates, defined as the time from diagnosis of malignancy to the first adverse event (death, second malignancy) or date of last follow-up; patients transplanted in the first remission were excluded from analysis of EFS (*n*=7). Cumulative incidence of malignancy (CIM) was determined as the proportion of patients who developed the first episode of cancer at a certain age. Analyses of OS, EFS, and CIM rates were performed using the Kaplan–Meier method and were compared using a log-rank (Mantel–Cox) test. The Fisher exact test was used to compare the incidence of malignancy in males and females. All statistics were calculated with GraphPad Prism 6 (GraphPad Software, La Jolla, CA, USA). Values of *p*<0.05 were considered significant.

## Results

### Patients’ Characteristics and Geographical Distribution

Our study cohort comprised 136 patients with NBS. Of these, 76 cases have not been previously published, and 60 are previously published but newly annotated cases (2, 13–17). One hundred thirty-six patients (63 males, 73 females) from 127 families were assessed, including 9 pairs of siblings and one family having two half-siblings (16). The majority of our cohort is unrelated individuals (*n*=109, 80%). As of February 1, 2019, 57 patients were deceased and 79 (59%) were alive. The age of the patients who were still alive ranged from 0.5 to 32 years (median age 11 years). Among all NBS patients, only 12 patients (8.9%) survived beyond 18 years of age.

The clinical diagnosis of NBS was confirmed by the identification of the pathogenic homozygous c.657_661del5 founder variant in the *NBN* gene in all subjects except one female patient from Russia who had a compound heterozygous with the combination of founder variants, c.657_661del5 and c.681delT (15).

A clinical description of our East Slavic NBS cohort is summarized in **Table 1**.

**Table 1:** Clinical description of Eastern Slavic cohort of NBS patients (*n*=136).

Country	Number of patients, gender	Period of birth	Death during follow-up	Malignancy (n, %)	Death due to malignancy (n, %)	Total HSCT / death after HSCT (n)
Ukraine	n=56 32M, 24F	1996-2017	23 (41%)	22 (39%)	15 (68%)	2/2
Russia	n=53 17M, 36F	1994-2017	23 (43%)	25 (48%)	13 (52%)	5/3
Latvia (Russian origin)	n=5 3M, 2F	1993-2012	2 (40%)	4 (80%)	2 (50%)	0
Belarus	n=22, 11M, 11F	1983-2017	7* (32%)	9 (41%)	6 (67%)	6/0

M, male, F, female, n, number.

*3 of 7 deceased patients were diagnosed postmortem.

The median age at diagnosis did not differ by countries (Ukraine: median 4 years [range 0.1–16], Russia: 5 years [range 0.2–14], Belarus: 3.55 years [range 0.3–21]) (**Figure S1A**, in this article’s Online Repository). Patients born in the capital or major cities in each Eastern Slavic country were diagnosed at an earlier age compared with those born in smaller cities or rural regions. Median age of diagnosis was lower in Minsk and the Minsk region (Belarus, **Figure S1C**) and in Moscow and the Moscow region (Russia, **Figure S1D**). In Ukraine, patients residing in the western part of the country, including Lviv and the Lviv region, were diagnosed at an earlier age (**Figure S1B**).

**Figure 1:**
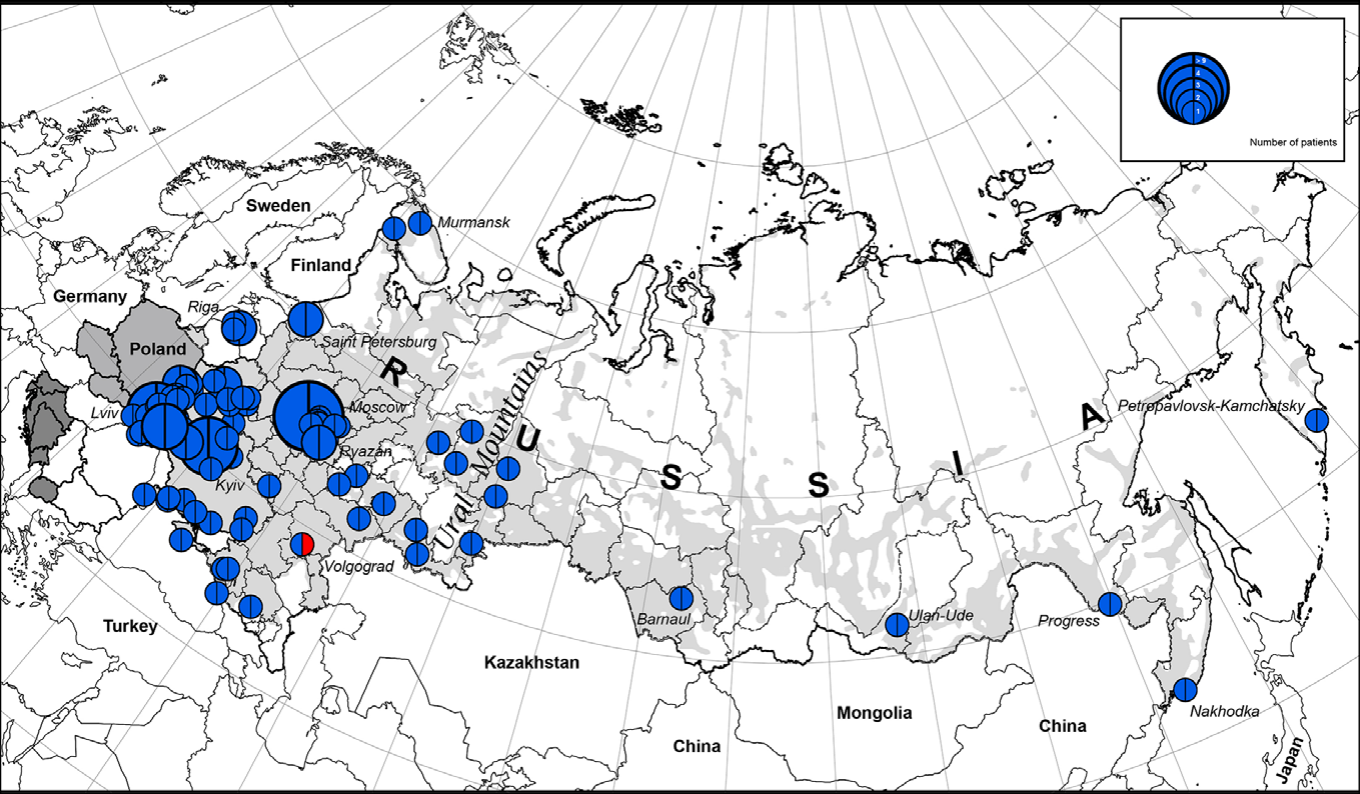
The geographic distribution of NBS patients among East Slavs. Light gray zone on the map of Russia is the territory of predominant Russian settlement (18). The size of the full circle correlates to the number of patients diagnosed from one location (or region) (range 1 to 9). In blue is the *NBN* founder variant c.657_661del5, p.K219fsX19; in red: *NBN* c.657_661del5/c.681delT.

We illustrate the geographic distribution of NBS patients among Eastern Slavs on the map of Eastern Europe and the Russian Federation (one patient from each family) (**Figure 1**).

**Figure 2:**
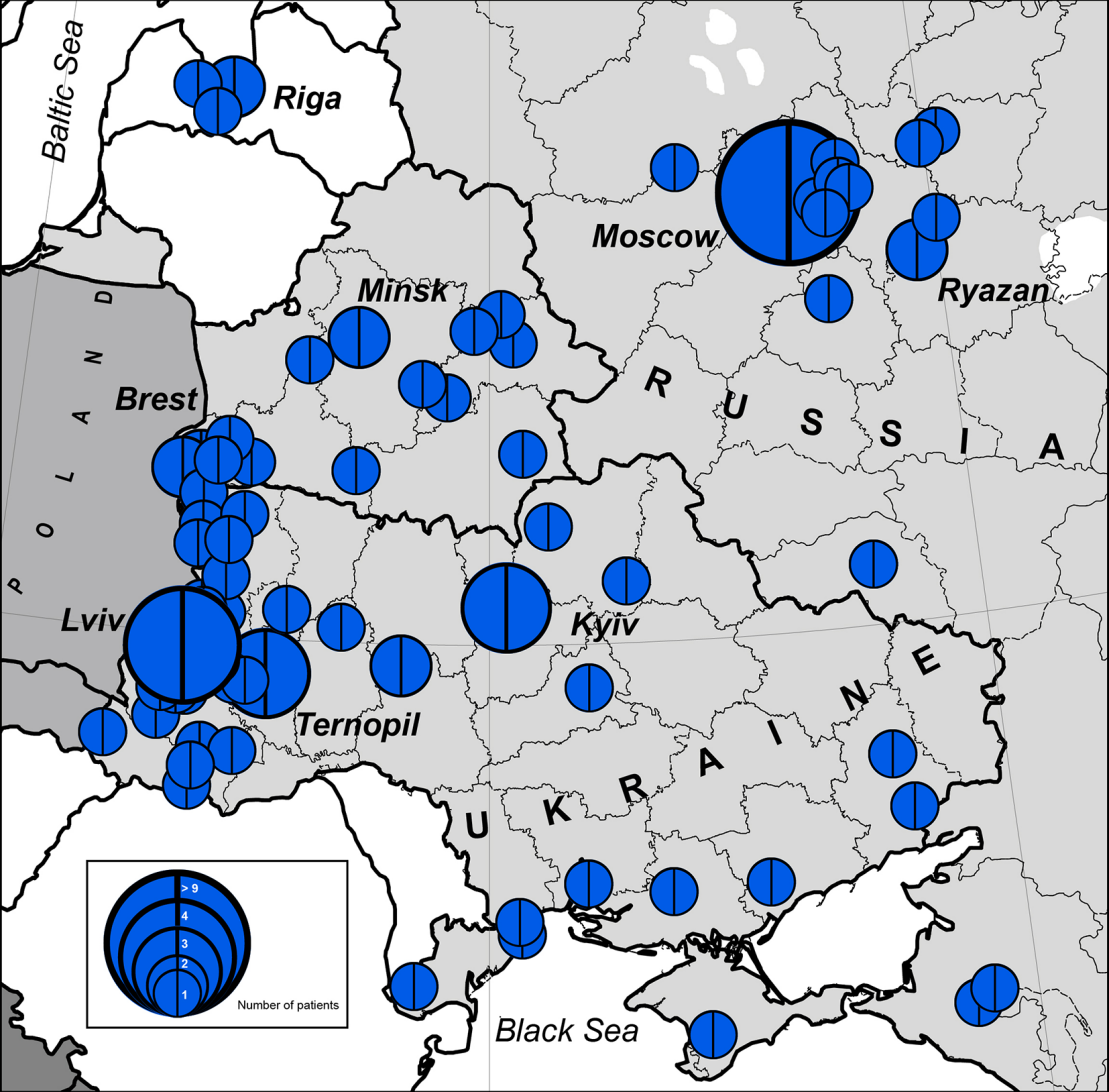
Number of patients with NBS in geographical hot spot region. In blue is the *NBN* founder variant c.657_661del5, p.K219fsX19. The size of the full circle correlates to the number of patients diagnosed from one location (or region). Blue circles represent number of cases (1 to >9).

The highest concentration of NBS patients was observed in the western part of Belarus (Brest region) and Ukraine (Lviv region and Ternopil), Latvia (Riga), and Russia (Moscow and nearby regions) (**Figures 1** and **2**).

The western parts of Belarus and Ukraine share their border with Poland, the country with the highest number of NBS patients diagnosed in the world (10). Identification of patients with NBS in Russia corresponds to resettlement of Russians in the Russian Federation (**Figures 1** and **2**).

### The Prevalence of NBS Patients Among Slavs

We estimate the prevalence of patients with NBS among Eastern and Western Slavs using data from our cohort as well as previously published reports (**Table 2**). Overall, our analysis shows a greater prevalence of NBS in Western Slavs. Specifically, Czech Republic (3.1 per 1,000,000, year 2016) (4) and Poland (3.1 per 1,000,000, year 2015) (10) had the highest prevalence of NBS patients, followed by Slovakia (2.6 per 1,000,000, year 2004). In the Eastern Slavs, Belarus had the highest prevalence of NBS (2.3 per 1,000,000, year 2018) (**Table 2**). The lower prevalence of NBS in Ukraine (1.3 per 1,000,000, year 2018) and Russia (0.7 per 1,000,000, year 2018) may reflect that the settlement of this area is not homogenous (**Figure 1**) although it should be noted that Slavic people are the biggest population in Russia (**Table 2**).

**Table 2:** Prevalence of NBS patients among Eastern and Western Slavs.

Country	Population (as of year studied)	Number of patients	Prevalence (per 1,000,000)	Reference
***East Slavs***				
Belarus	9,500,882 (2018)	22	2.3	Current study
Russia	118,764,737* (2018)	81	0.7	Current study and (9)
Ukraine	42,216,766 (2018)	56	1.3	Current study
***West Slavs***				
Poland	38,437,239 (2015)	118	3.1	(10)
Czech Republic	10,597,473 (2016)	33	3.1	(4)
Slovakia	5,372,343 (2004)	14	2.6	(4)

*80.9% are Russians among 146,804,372 population of the Russian Federation.

We estimated the period prevalence of NBS over a 20-year period, comparing the western and eastern regions of the countries under study (**Table S1**). Our analysis shows that the western part of Belarus and Ukraine represent the geographical center of NBS patients among Eastern Slavs (**Figure 2**). The period prevalence of NBS patients ranged from 6.4 per 1,000,000 (Ivano-Frankovsk) to 24 per 1,000,000 (Lviv) in West Ukraine—a territory that covers an area of 131,256 km^2^ (8 regions, **Table S1**) with a population of 9,765,000 (year 2019) and 35 patients. The prevalence in West Ukraine was estimated to be 3.6 per 1,000,000. Similarly, in the western region of Belarus, where the highest prevalence was estimated to be 21 per 1,000,000 (Brest) (**Table S1**), the prevalence of NBS is higher than that in Poland and Czech Republic (**Table 2**). Collectively, these data indicate that the western parts of Ukraine and Belarus could be established as an “East Slavic NBS hot spot and geographical center.”

### Demographic and Treatment Characteristics of Patients With Malignancy

**Figure 3:**
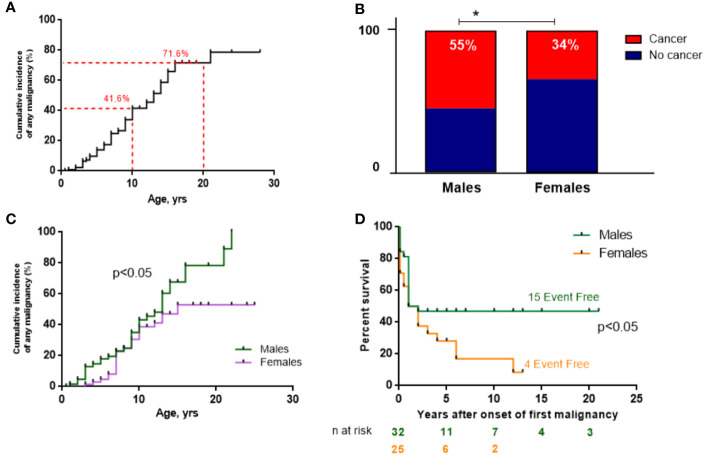
Malignancy data of the NBS East Slavic cohort. **(A)** Cumulative incidence of any malignancy (%) from birth in the whole NBS East Slavic cohort. **(B)** Percentage of developed malignancy in boys and girls with NBS. **(C)** Cumulative incidence of any malignancy (%) among boys and girls in the NBS East Slavic cohort. **(D)** EFS displayed as Kaplan–Meier survival curves in males and females after first malignant diagnosis. *p < 0.05.

Malignancy occurred in 62 (45.6%) patients with NBS. The CIM by the age of 10 years amounted to 41.6% (*n*=44/105) and dramatically increased further to 71.6% (*n*=18/25) by the age of 20 years (**Figure 3A**). The male/female (M:F) ratio in patients with cancer was approximately 1.5:1 (37 M and 25 F) although the entire NBS cohort had a slight female predominance (1:1.2).

Malignancy developed significantly more often in males (55.2%) than in females with NBS (34.2%) (*p*=0.017; **Figure 3B**) with a 26% increase in CIM for males by the age of 20 years compared to females (79.2% vs. 52.9%, *p*=0.047) (**Figure 3C**). However, those females with cancer had worse EFS rates than males (long-term EFS 16.6% vs. 46.8%, *p*=0.036; **Figure 3E**). The difference was most prominent after puberty (>14 years old). Currently, 21 patients are alive (33.9%, 3 of 21 after HSCT), and the median follow-up is 17 months (range 0–248 months) for the whole group of 62 NBS patients with malignancy.

Lymphoid neoplasms (91.9%) represent the vast majority of malignancies with a slight predominance of T-lineage malignancies (54.9%). Non-Hodgkin lymphomas (NHL) were the most frequent disease and comprised about 2/3 (42 out of 62) of all cases. The most common NHL subtypes were diffuse large B-cell lymphoma (DLBCL) (*n*=16, 38.1%) and T-lymphoblastic lymphoma (T-LBL) (*n*=13, 31.0%), followed by peripheral T-cell lymphoma (PTCL) (*n*=5, 11.9%), Burkitt lymphoma (*n*=2, 4.8%), B-cell NHL unspecified (*n*=2, 4.8%), and systemic ALK-positive anaplastic large cell lymphoma in 1 case (2.4%). No data on NHL subtype or stage were available in 3 cases. In all provided NHL cases except one, the patients had an advanced stage of the disease (Murphy stage III: 25, IV: 13). Remarkably, 75% of patients with DLBCL showed lung involvement. Nine out of 13 with T-LBL had generalized stage IV disease with bone marrow involvement.

Hodgkin lymphoma (HL) appeared to be a rare malignancy in NBS patients with only 2 (3.3%). Both of them were diagnosed at a relatively early age (5.7 and 6.6 years, respectively) in an advanced stage (Ann-Arbor stage IV) and showed quite aggressive histology (mixed cellularity and lymphocyte depleted subtype, respectively).

A total of 12 cases of acute lymphoblastic leukemia (ALL) were diagnosed in our cohort with only one of B-lineage differentiation (common-B immunophenotype). Nine ALL cases were of T-lineage origin with the following immunophenotypes: ETP-ALL in 1 case, cortical in 3, pre-T in 3, and T-mature (CD8+) in 2. Immunophenotyping data were not provided in 2 ALL cases. Two other patients with acute leukemia were diagnosed: one with acute biphenotypic (early T/early myelo) and one with acute myeloid leukemia.

Solid tumors appeared to consist of a significant minority in the cancer spectrum in the NBS cohort with only 4 (6.5%) revealed cases (**Table S2**).

Given the retrospective and multi-institutional nature of the study, detailed information regarding treatment was scarce. Five patients did not receive special treatment (2 refused and 3 died before therapy). Data about the treatment of 8 other patients were not provided. Forty-nine patients were treated according to ongoing protocols (*n*=44) or individualized (*n*=5) and analyzed for treatment results. Complete remission (CR) was achieved in 35 (71.4%) treated patients. Results varied depending on histology and were studied in 12 out of 13 patients with T-LBL, 10 out of 12 cases with ALL, 10 out of 15 cases with DLBCL, and 0 of 4 patients with PTCL. It should be noted that all 3 patients with T-mature ALL/LBL responded poorly on induction chemotherapy but managed to achieve CR after 1–3 high-risk courses of treatment. All 4 patients with PTCL showed disease progression at different stages of treatment. Nine (18.4%) deaths occurred before CR or before recurrence occurred (7 during induction therapy and 2 later), and 5 deaths occurred during remission because of infections, bleeding, or multiple organ dysfunction.

Disease recurrence occurred in 12 (24.5%) patients, including progression in 7 and relapse in 5 cases. Median time to disease recurrence was 8 months (range 1–67 months). No one achieved second remission or survived.

A second malignancy occurred in 4 out of 21 (19.0%) nontransplanted patients, who had a follow-up more than 3 years after the first cancer diagnosis. The first tumor was NHL in all cases (DLBCL in 3 and T-LBL in 1). We observed 1 case of medulloblastoma and 3 cases of the second lymphoma, diagnosed in 11.9, 4.4, 4.5, and 10.2 years, respectively after the first malignancy. All 4 patients who developed the second malignancy died due to various reasons.

**Figure 4:**
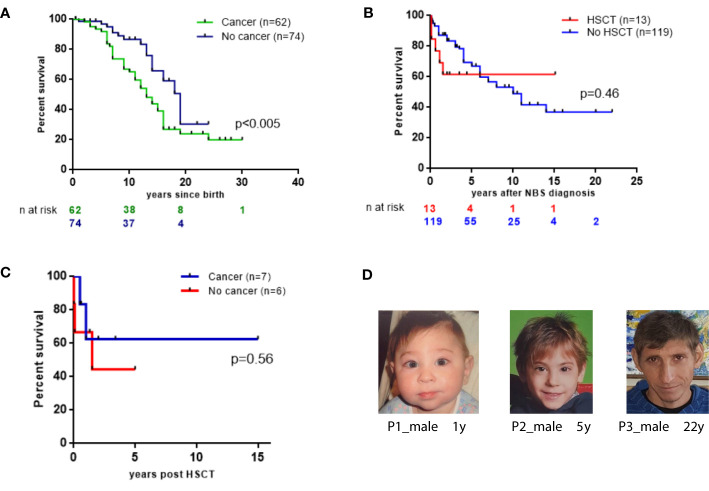
Survival probabilities of 136 East Slavic NBS. **(A)** OS displayed as Kaplan–Meier survival curve of cancer (+) and no cancer NBS patient groups. **(B)** The probability of the OS since NBS diagnosis in 13 patients who underwent HSCT and in 119 nontransplanted patients (expressed as a percentage, patients diagnosed postmortem were excluded from analysis). **(C)** Survival probabilities of 13 NBS patients after HSCT. **(D)** NBS patients’ appearance at different ages.

Patients who developed cancer in our cohort had a lower 20-year OS than those without malignancy (23.9% vs. 30.4%; *p*=0.0078 **Figure 4A**).

### Allogeneic HSCT

Among 136 NBS patients, HSCT was performed in 13 patients (9.5%; 8 males and 5 females) (**Table S3**). The median age at HSCT was 12.3 years (range, 1.9–22.1). The stem cell source was bone marrow (BM) in 10 patients. The donor was an unrelated HLA match in 10 cases, and 3 cases were HLA-matched siblings.

Indication for HSCT was malignancy in 7 patients (in the first CR in 4, in the second CR in 1, and after the second lymphoma in 2) and immunodeficiency in 6 cases (2 patients were transplanted preemptively with mild signs of recurrent infections at the age of 1.9 and 2.2 years; 4 patients had chronic, severe infections of the upper and lower respiratory tract, and 1 out of 4 additionally had skin granuloma). Four patients with leukemia/lymphoma who had undergone HSCT in the first CR are alive without evidence of malignancy (follow-up period ranged from 11 months to 5.3 years). One patient with a relapse of ALL received myeloablative conditioning and died soon after HSCT. All the others (91.7%) received reduced-intensity conditioning (RIC) regimens (**Table S3**). Three patients who were transplanted after the treatment of relapsed ALL or second lymphoma, died of sepsis and multiorgan failure before hematopoietic recovery or after relapse of malignancy.

Of the 13 patients who underwent HSCT, acute graft-versus-host disease (GVHD) was present in 5 (38.5%) patients and chronic GVHD in 4 patients. Viremia caused by adenovirus, cytomegalovirus, Epstein-Barr virus, BK virus or a combination was reported in 5 patients. None of the transplant patients fully rejected the graft.

HSCT after the RIC regimen appeared to be tolerable and effective in NBS patients. Having 1 CR and a good performance status at the time of HSCT are probably predictive of better outcomes.

From the time of NBS diagnosis, the probability of 15-year OS in patients who underwent HSCT was 61.5%. In contrast, patients who did not undergo HSCT had a 15-year OS of 36.9% (**Figure 4B**). In total, 8 (61.5%) NBS patients are alive after HSCT without *de novo*, recurrent, or second malignancies after a median length of follow-up for 41 months (range 11–169 months). Among the 6 patients who underwent HSCT and did not have malignancy, 2 died of complications after HSCT (**Table S3**, **Figure 4C**).

### Social Adaptation and QoL in Adult NBS Patients

Of the 12 adult survivors in our cohort, 11 (6 males and 5 females) responded to our survey (2 male patients were transplanted 14 and 5 years ago; their QoL scores were the same as in patients who experienced malignancy many years ago). The median age of these patients was 24.6 years (range 18–32). Nine patients indicated some degree of independence (i.e., ability to take care of their routine needs). One female patient had her own family, and all others lived with their parents. All patients were able to attend school with their healthy peers and completed their elementary school education. Two male patients graduated from college but could not work in their profession. One female patient graduated from a university and worked as a psychologist with neurologically disabled and autistic children. She was the only responder who enjoyed her job and had an active hobby. This was also the only patient who reported strong social support from her parents for education and socialization. In total, 6 of 11 young adult NBS patients were employed. Six of the 11 respondents confirmed socialization with peers.

We further evaluated patients’ QoL using the SF-36 Health Survey. Our analysis shows that adult NBS patients had substantially impaired QoL in both the physical and mental domains (**Figure S2**). The subanalysis demonstrates remarkably lower self-reported scores in physical functioning, role–physical, bodily pain, and general health for most patients as well as in vitality, social functioning, role–emotional, and mental health. The overall role–physical (<25) and bodily pain and MCS (<23) scores were remarkably lower (the healthy population has >83 scores as very good QoL [12]) (**Figure S2**) in patients who experienced cancer in the last 1 to 2 years of life compared with patients who experienced cancer in childhood.

Our study shows that adult NBS patients find substantially impaired HRQoL in the physical and mental domains.

## Discussion

In our study, we present the largest East Slavic cohort of patients with NBS retrospectively collected over a 35-year period (1983–2018). Our analysis demonstrates a higher prevalence of NBS in the western parts of Ukraine and Belarus, particularly in regions bordering Poland. The presence of this East Slavic NBS geographical hot spot is consistent with our previous study of *RAG* founder variants in Slavs, which revealed the Vistula watershed as the geographic center of the presumed origin of the p.K86fsTer33 *RAG1* pathogenic variants (19). Further studies are needed to understand the drivers behind this trend and to address the needs of these patients.

A Slavic homozygous c.657_661del5 variant in the *NBN* gene was found in all patients except one. A heterozygous compound c.657_661del5/c.681delT was detected in a female patient from Russia; the only reported case so far in the Slavic population (17).

Although NBS patients have characteristic features (**Figure 4D**), immune and genetic evaluation was not initiated for all patients early in life. Therefore, diagnosis of these children was delayed and occurred in the context of malignancies. Consequently, other preventive measures, such as intravenous immunoglobulin replacement therapy, was delayed, which, in turn, increased patients’ susceptibility to infections and contributed to challenges in their management.

Consistent with previous studies, our study shows that 40% to 60% of NBS patients develop lymphoma or leukemia by the age of 20 years (5, 9, 10). We observed 46% frequency of malignancy in patients with NBS by the end of our follow-up period with mostly lymphoid tumors (92%). However, in contrast with published data (10), our cohort showed a slight predominance of T-cell malignancies.

Survival rates of NBS patients with cancer were lower in comparison with immunocompetent patients in all reports and ranged from 18% to 40% (9, 10). Attention and awareness of pediatricians may expedite immunological and molecular genetic testing for children with suspected NBS and promote monitoring for development of malignancy. According to our data, the risk of tumor development in males with NBS is higher than in females (65% and 35%, respectively, **Figure 3B**). However, the survival rate of females with tumors without HSCT is significantly worse (17% vs. 47%, **Figure 3D**) and may be partly due to the higher proportion of prognostically unfavorable large cell lymphomas in females compared with males. Larger studies are needed to determine the predictive value of gender in NBS patients with malignancy. Patients who developed cancer in our cohort had a lower 20-year OS than those without malignancy (23.9% vs. 30.4%; *p*=0.0078; **Figure 4A**). Our data are strikingly different from a previous Polish study (10) that reported a 20-year OS of more than 80% in NBS patients without cancer. Heterogeneity in patient characteristics and treatments may have contributed to this inconsistency. Our cohort comprised a higher number of younger patients. Furthermore, poor medical treatment in adult patients with severe complications caused by an underlying immunodeficiency may have led to poorer survival outcome in our cohort.

Taking into account a progressive immunodeficiency with deteriorating clinical state and extremely high risk of first and subsequent lymphoid malignancies with uncertain curative potential, allogeneic HSCT with RIC may be a rational therapeutic option for NBS patients with severe defects of immune function and for any NBS patients with lymphoid malignancy in first remission (14). However, the biggest Russian NBS study group reported that an RIC was a reason for a high level of graft failure and mixed chimerism in NBS patients (20).

According to guidelines from the Inborn Errors Working Party of the European Blood and Marrow Transplant Society, transplantation is recommended for all NBS patients in first CR of lymphoma or leukemia (21). In our study, a large fraction of the patients received HSCT for indication of underlying immunodeficiency (6/13) and 67% (4/6) survived. This is a unique group and their cancer-free survival outcome will be followed in our future studies. Nevertheless, there is still not enough data to recommend HSCT in all NBS even with significant cellular immunodeficiency but without clinical symptoms of immune deficiency (21).

QoL studies are scarce for patients with DNA repair defects although the risk of malignancy and neurological sequelae are very high in these groups. Current publications in patients with ataxia telangiectasia focus on pulmonary and gastrointestinal interventions and their effect on QoL (22, 23); however, social and psychological assessment and HRQoL surveys are lacking in the literature. This is of high importance as these assessments could justify programs to advocate for children and their families with NBS in hot spot regions.

Our very limited long-term study shows rare survival up to 30 years of age even without HSCT, and many of the patients had poor social adaptation and QoL of different degrees. Patients’ QoL strongly depended on family support as well on medical and social care.

Overall, our geographical studies show an East Slavic hot spot region with a high prevalence of patients with NBS founder mutation. Malignancies of lymphoid origin were frequent, and HSCT was attempted to improve their outcome. In addition to the high risk of malignancy, patients with NBS experience psychosocial challenges, and interventions are needed to promote their QoL. Genetic counseling and screening before family planning should be considered for those with family history of this disease, especially in the hot spot region.

## Data Availability Statement

The original contributions presented in the study are included in the article/**Supplementary Material**. Further inquiries can be directed to the corresponding author.

## Ethics Statement

The protocol of study was approved by the institutional review board of Belarusian Research Center for Pediatric Oncology, Hematology and Immunology (IRB protocol #0011715). Written informed consent to participate in this study was provided by the participants’ legal guardian/next of kin. Written informed consent was obtained from the individual(s), and minor(s)’ legal guardian/next of kin, for the publication of any potentially identifiable images or data included in this article.

## Author Contributions

SS designed the research, collected data, interpreted and analyzed the results. SS, M-SO and JW wrote the manuscript. AF and SS wrote malignancy section. DV prepared the maps. OP, AVB, SV, TP, AF, IS, YM, TV, AP, IT, EV, ISS, EP, NR, IN, SK, SA, EG, NM, MB, EL, TL, AGB, HA, HM, OK, IK, LK, and OA provided patient’s information. EL, TP, IS, ISS and TV collected QoL data, MB and SS analyzed and wrote QoL section. M-SO revised epidemiologic data. JW, IK, LK, and OA guided the writing of the manuscript. All authors contributed to the article and approved the submitted version.

## Conflict of Interest

The authors declare that the research was conducted in the absence of any commercial or financial relationships that could be construed as a potential conflict of interest.
