# Supplementary material for: Geographical Distribution, Incidence, Malignancies, and Outcome of 136 Eastern Slavic Patients With Nijmegen Breakage Syndrome and NBN Founder Variant c.657_661del5
Source: Front Immunol. 2021 Jan 8;11:602482. doi: 10.3389/fimmu.2020.602482 (PMC7819964; doi:10.3389/fimmu.2020.602482)
Supplement: Supplementary file 1 [file DataSheet_1.docx]

|  |  |  |  |  |  |  |  |  |
| --- | --- | --- | --- | --- | --- | --- | --- | --- |
|  | **Table S1. Estimated period prevalence of NBS patients in Belarus and Ukraine** | | | | | | |  |
|  |  |  |  |  |  |  |  |  |
|  | **Country/region** | **20-years period** | **Sum of newborns for the period for the 20-yrs period** | **Number of NBS patients for the 20-yrs period** | **Period prevalence per 1,000,000** | **Whole period** | **Number of NBS patients during whole period** |  |
|  |  |  |  |  |  |  |  |  |
|  | **BELARUS** |  |  |  |  |  |  |  |
|  | ***Western regions*** |  |  |  |  |  |  |  |
|  | Brest region | 1998-2017 | 329 608 | 7 | **21.2** | 1983 – 2014 | 11 |  |
|  | Minsk region | 1998-2017 | 397 600 | 2 | 5.0 | 1996 – 2017 | 3 |  |
|  | Grodno region | 1998-2017 | 239959 | 0 | **--** | 1994 | 1 |  |
|  |  |  |  |  |  |  |  |  |
|  | ***Eastern regions*** |  |  |  |  |  |  |  |
|  | Mogilev region | 1998-2017 | 231 694 | 4 | 17.3 | 2004 - 2014 | 5 |  |
|  | Vitebsk region | 1998-2017 | 236 504 | 0 | **--** | **--** | 0 |  |
|  | Gomel region | 1998-2017 | 318 186 | 2 | 6.3 | 1989 - 2007 | 2 |  |
|  |  |  |  |  |  |  |  |  |
|  |  |  |  |  |  |  |  |  |
|  | **UKRAINE** |  |  |  |  |  |  |  |
|  | ***Western regions*** |  |  |  |  |  |  |  |
|  | Volinska region | 1998-2017 | 267 475 | 4 | 15.0 | 1996 - 2015 | 5 |  |
|  | Zakarpat’e region | 1998-2017 | 327 278 | 3 | 9.2 | 1994 - 2016 | 3 |  |
|  | Ivano-Frankovsk region | 1998-2017 | 310 422 | 2 | 6.4 | 2002 - 2004 | 2 |  |
|  | Lviv region | 1998-2017 | 542 464 | 13 | **24.0** | 1989 - 2016 | 17 |  |
|  | Rivno region | 1998-2017 | 312 844 | 1 | **--** | 1998 | 1 |  |
|  | Ternopil region | 1998-2017 | 224 567 | 5 | **22.3** | 1995 - 2017 | 6 |  |
|  | Khmelnitski region | 1998-2017 | 268 131 | 1 | **--** | 2003 | 1 |  |
|  | Chernovtsi region | 1998-2017 | 205 991 | 0 | **--** | **--** | 0 |  |
|  |  |  |  |  |  |  |  |  |
|  | ***Central regions*** |  |  |  |  |  |  |  |
|  | Chernihiv region | 1998-2017 | 185 429 | 2 | 10.8 | 2009-2012 | 2 |  |
|  | Cherkasy region | 1998-2017 | 228 436 | 1 | **--** | 2007 |  |  |
|  | Kiev | 1998-2017 | 566 162 | 4 | 7.2 | 2003-2012 | 4 |  |
|  | Vinnitsa region | 1998-2017 | 326 689 | 2 | 6.1 | 2015 | 1 |  |
|  |  |  |  |  |  |  |  |  |
|  | ***Southeast regions*** |  |  |  |  |  |  |  |
|  | Kherson region | 1998-2017 | 224 157 | 1 | **--** | 2006 | 1 |  |
|  | Donetsk region | 1998-2017 | 683 934 | 1 | **--** | 1994-2000 | 2 |  |
|  | Odessa region | 1998-2017 | 508 023 | 3 | 5.9 | 2005-2016 | 3 |  |
|  | Mykolaiv region | 1998-2017 | 233 275 | 1 | **--** | 2003 | 1 |  |
|  |  |  |  |  |  |  |  |  |
|  |  |  |  |  |  |  |  |  |
|  |  |  |  |  |  |  |  |  |

|  |  |  |  |  |  |  |  |  |  |
| --- | --- | --- | --- | --- | --- | --- | --- | --- | --- |
|  |  | **Table S2. Characteristics and probability of event-free survival (EFS) of NBS patients with malignancy** | | | | | | | |
|  |  |  |  |  |  |  |  |  |  |
|  |  | **Characteristic** | **Patients,  n (%)** | **Events, n** | **10y EFS,  %** | **20y EFS,  %** | **Log-rank р** |  |  |
|  |  | All patients | 62 (100) | 41 | 32.8+6.6 | 24.6+7.1 |  |  |  |
|  |  | Age: |  |  |  |  |  |  |  |
|  |  | < 10 years | 40 (64.5) | 26 | 33.6+8.5 | 22.4+10.8 | 0.75 |  |  |
|  |  | > 10 years | 22 (35.5) | 15 | 31.2+10.8 | 24.9+10.3 |  |  |  |
|  |  | Sex: |  |  |  |  |  |  |  |
|  |  | male | 37 (59.7) | 21 | 43.6+8.6 | 34.9+10.4 | 0.12 |  |  |
|  |  | female | 25 (40.3) | 20 | 32.8+6.6 | 24.6+7.1 |  |  |  |
|  |  | Type of malignancy: |  |  |  |  |  |  |  |
|  |  | NHL | 42 (67.7) | 26 | 40.1+8.0 | 30.0+8.6 | 0.92 |  |  |
|  |  | HL | 2 (3.2) | 2 | – | – |  |  |  |
|  |  | ALL | 12 (19.4) | 7 | 0 | 0 |  |  |  |
|  |  | ABL | 1 (1.6) | 1 | – | – |  |  |  |
|  |  | AML | 1 (1.6) | 1 | – | – |  |  |  |
|  |  | Medulloblastoma | 1 (1.6) | 1 | – | – |  |  |  |
|  |  | Rabdomyomarcoma | 1 (1.6) | 1 | – | – |  |  |  |
|  |  | other | 2 (3.2) | 2 | – | – |  |  |  |
|  |  | Stage: |  |  |  |  |  |  |  |
|  |  | II | 1 (1.6) | 1 | – | – |  |  |  |
|  |  | III | 25 (40.3) | 19 | 26.7+9.1 | 17.8+9.4 | 0.20 |  |  |
|  |  | IV | 16 (25.8) | 9 | 44.4+14.2 | 29.6+15.3 |  |  |  |
|  |  | Leukemia | 14 (22.6) | 7 | 0 | 0 |  |  |  |
|  |  | No data | 6 (9.7) | 5 | – | – |  |  |  |
|  |  | Treatment: |  |  |  |  |  |  |  |
|  |  | Protocol | 44 (71.0) | 25 | 40.9+8.6 | 27.2+9.7 | 0.036* |  |  |
|  |  | Individual | 5 (8.1) | 5 | 0 | 0 |  |  |  |
|  |  | No treatment | 5 (8.1) | 5 | 0 | 0 |  |  |  |
|  |  | No data | 8 (12.9) | 6 | 25.0+15.3 | 25.0+15.3 |  |  |  |
|  |  | Allogeneic HSCT: |  |  |  |  |  |  |  |
|  |  | yes | 6 (9.7) | 3 | 40.0+29.7 | 0 | 0.18 |  |  |
|  |  | no | 56 (90.3) | 38 | 30.8+6.7 | 26.4+7.0 |  |  |  |
|  |  |  |  |  |  |  |  |  |  |
|  |  |  |  |  |  |  |  |  |  |
|  |  |  |  |  |  |  |  |  |  |
|  |  |  |  |  |  |  |  |  |  |
