# Supplementary material for: Geographical Distribution, Incidence, Malignancies, and Outcome of 136 Eastern Slavic Patients With Nijmegen Breakage Syndrome and NBN Founder Variant c.657_661del5
Source: Front Immunol. 2021 Jan 8;11:602482. doi: 10.3389/fimmu.2020.602482 (PMC7819964; doi:10.3389/fimmu.2020.602482)
Supplement: Supplementary file 2 [file DataSheet_2.pdf]

Table S3. Characteristics of HSCT in NBS patients

N Country of origin	Gender	Country of HSCT	Age by HSCT (years)	Indication	Donor/stem cell source	Conditioning	GVHD prophylaxis	aGvHD	Complications	cGVHD	Follow-up	Outcome
1_Belarus	M	Belarus	13.3	Immunodeficiency	MSD / BM	Bu 8mg/kg Flu 150mg/m2 ATG	CSA, MTX	Grade 2, skin	Mucositis, dermatitis	Ext, skin, liver	14.1 years	Alive
2_ Belarus	F	Belarus	6.8	Immunodeficiency	MUD / BM	Flu 150 mg/m2 Cy 20 mg/kg ATG 7.5 mg/kg Rituximab 200 mg/m2	CSA, MMF	no	Dermatitis, BKV, JCV acivation, Toxo-infection	no	2.5 years	Alive
3_Ukraine	F	Poland	14.3	Immunodeficiency	9/10 MUD / BM	Flu Cy ATG	CSA, MMF	Grade 2, skin	Hemophagocytic complication, cardiomyopathy, EBV infection reactivation, bronchiolitis	no	1.1 years	Died of sepsis
4_Ukraine	M	Turkey	5.0	Immunodeficiency	9/10 MUD / BM	Flu 150mg/m2 Cy 40 mg/kg ATG 60 mg/kg	ND	Grade 3 skin, intestine	CMV, pneumonia	no	6.5 months	Died of sepsis
5_Russia	F	Russia	1.9	Immunodeficiency	MUD / BM	Cy 20 mg/kg Bu 4 mg/kg Flu 150 mg/m2 ATG 10 mg/kg Rituximab 200 mg/m2	MTX, tacrolimus	Grade 2, skin	Mucositis, CMV viremia, hepatitis, enterocolitis	Grade 3, liver	3.4 years	Alive
6_Belarus	M	Belarus	2,16	Immunodeficiency	MSD / BM	Flu 150 mg/m2 Cy 20 mg/kg ATG 15mg/kg (F)	CSA,Med	no	dermatitis	no	7 months	Alive

7_Belarus	M	Belarus	22.1	Malignancy (T-mature ALL, high risk, 1CR)	MUD / BM	Flu 150 mg/m2 Mel 140 mg/m2 ATG 60 mg/kg	CSA, MMF	Grade 4, skin, gut	Mucositis, pancreatitis, toxic hepatitis, hemorrhagic cystitis, sepsis, CMV, BKV, Rota, Astro infections	Ext, gut, eyes	4.8 years	Alive
8_Belarus	M	Belarus	5.3	Malignancy (T-mature LBL, high risk, 1CR)	MUD / BM	Flu 125 mg/m2 Mel 140 mg/m2 ATG 60 mg/kg	CSA, MTX	no	Mucositis, pancreatitis, enterocolitis	no	5.3 years	Alive
9_Belarus	M	Belarus	4.3	Malignancy (T-cortical ALL, intermediate risk, 1 CR)	MSD / BM	Flu 125mg/m2 Mel 140 mg/m2 ATG 60 mg/kg	CSA, MMF	no	Dermatitis, pancreatitis, toxic hepatitis, enterocolitis	no	11 months	Alive
10_Russia	F	Russia	12.3	Malignancy (second NHL, CR)	MUD / BM	RIC	ND	ND	ND	no	1 month	Died of sepsis
11_Russia	M	Russia	13.5	Malignancy (second T-NHL, CR)	MUD / PBSC	Bu 2mg/kg Flu 150mg/m2 Cy 20 mg/kg ATG 760 mg/kg Rituximab 200 mg/m2	Tacrolimus	no	Mucositis, dermatitis	Grade 2, liver	1.5 years	Died of progression after relapse
12_Russia	F	Russia	15.0	Malignancy (BL, 1CR)	MUD / PBSC	Bu 2mg/kg Flu 150mg/m2 Cy 20 mg/kg ATG 60 mg/kg Rituximab 200 mg/m2	Tacrolimus	no	Mucositis, dermatitis	no	1.3 years	Alive
13_Russia	M	Russia	16.0	Malignancy (relapse of T-ALL, 2CR)	MUD / PBSC	Cy 120 mg/kg TT 750 mg/m2 Bu 10 mg/kg	CSA, MTX	no	Mucositis, multiple organ failure, Intestinal obstruction, day +6		6 days	Died of sepsis

Abbreviations:

M - male
F - female
ALL - acute lymphoblastic leukemia; Astro - Astrovirus; ATG, Anti-thymocyte globulin; BKV, BK virus; BL - Burkitt lymphoma; BM - bone marrow; BMF, bone marrow failure; Bu, busulphan; CMV, cytomegalovirus; CSA, Cyclosporine A; Cy, cyclophosphamide; cGVHD – chronic Graft versus Host Disease; ext - Extensive chronic GVHD; CR - complete remission; Flu - fludarabine; GI - gastrointestinal; HSCT - hematopoietic stem cell transplantation; LBL - lymphoblastic lymphoma; Mel - Melphalan; MFD - matched family donor; MMF- mycophenolate mofetil; MMFD - mismatched family donor; MOF - multiorgan failure; MTX - Methotrexate; MUD - matched unrelated donor; NA - not available; NHL - non-Hodgkin lymphoma; OKT3 - Muromonab-CD3; PBSC - peripheral blood stem cells; Rota -Rotavirus; T-NHL - T-cell non-Hodgkin lymphoma; Treo - treosulfan; VOD - venoocclusive disease; Toxo - toxoplasma;
