# Supplementary material for: Geographical Distribution, Incidence, Malignancies, and Outcome of 136 Eastern Slavic Patients With Nijmegen Breakage Syndrome and NBN Founder Variant c.657_661del5
Source: Front Immunol. 2021 Jan 8;11:602482. doi: 10.3389/fimmu.2020.602482 (PMC7819964; doi:10.3389/fimmu.2020.602482)
Supplement: Supplementary file 3 [file DataSheet_3.docx]

**Supplemental data**

Figure S1.

| **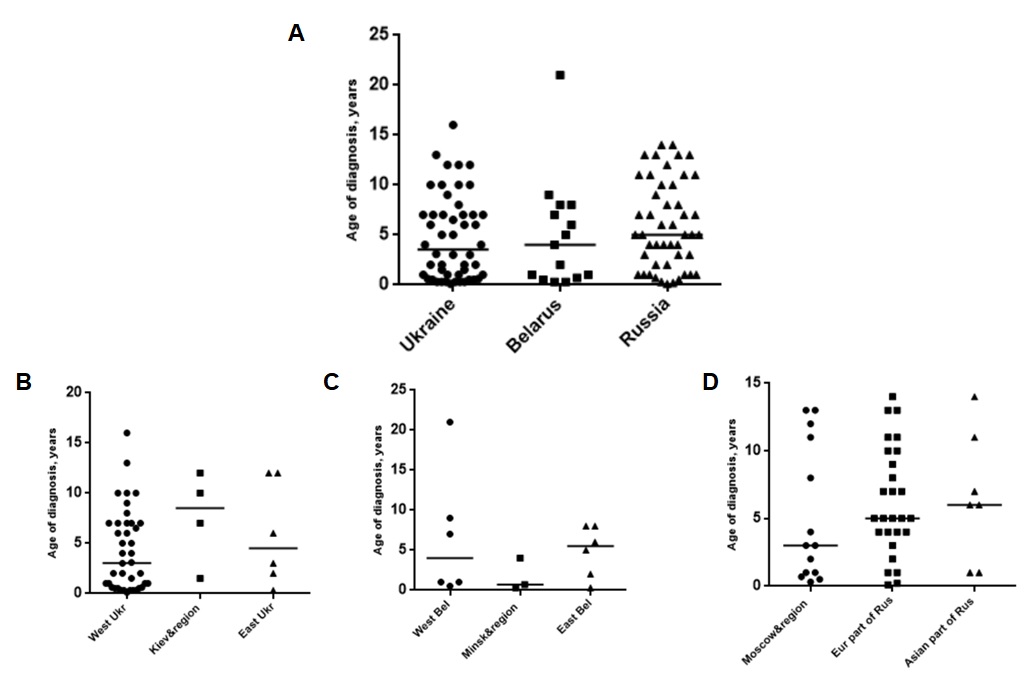** |
| --- |
| **FIGURE S1** \| **Age of NBS diagnosis in East Slavic countries.** **(A)** Median of age of NBS diagnosis in Ukraine, Belarus and Russia; **(B)** in Ukrainian regions; **(C)** in Belarusian regions; **(D)** Median age of NBS diagnosis in Russia, in European and Asian part of Russia. |

Figure S2.

| 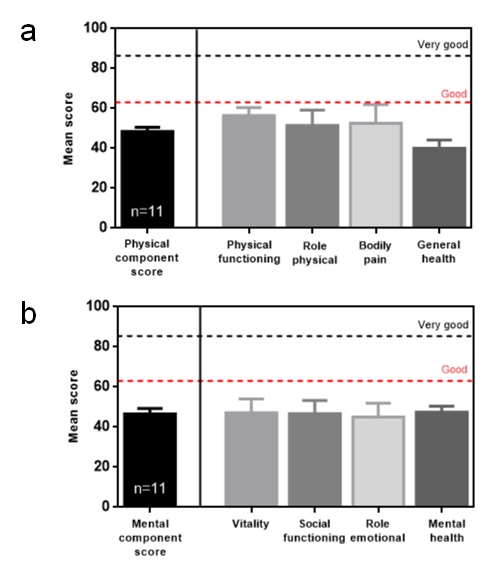 |
| --- |
| **FIGURE S2** \| **SF-36 physical (a) and mental (b) component and domain scores of adult NBS patients** at age older 18 years old (n=11). **a**. Physical functioning, role physical, bodily pain, and general health are domains of the physical component scores; vitality, social functioning, role emotional, and mental health are domains of the mental component score. ­­­ SF-36 mean current health scores for healthy population [adapted from 12]. |
